# Supplementary material for: Content-rich biological network constructed by mining PubMed abstracts
Source: BMC Bioinformatics. 2004 Oct 8;5:147. doi: 10.1186/1471-2105-5-147 (PMC528731; doi:10.1186/1471-2105-5-147)
Supplement: Additional File 2 — The original results of the above study (non-essential files are deleted to keep the file size under the limit set by BMC bioinformatics). [file 1471-2105-5-147-S2.bz2 › chilibotAdditionalFile2/dip50/658ID10525412E11049/html/NIFD_NIFK.html]

 


 **NIFD** and **NIFK** 
  
Found 195 abstracts in PubMed, retrieved 50.  
 

 What does Google say? 
 PDF only 
| .edu only 

---

**Interactive relationship** (e.g. stimulation, inhibition, etc)

**Neutral relationship**- this change did not have a substantial effect on the interaction between dinitrogenase  [ **NIFK** ]   [ **NIFD** ]  reductase and DRAT.  Ref: 10762264 J Bacteriol, 2000
- Dinitrogenase  [ **NIFK** ]   [ **NIFD** ]  reductase is posttranslationally regulated by dinitrogenase  [ **NIFK** ]   [ **NIFD** ]  reductase ADP ribosyltransferase DRAT via ADP ribosylation of the arginine 101 residue in some bacteria.  Ref: 11114923 J Bacteriol, 2001
- The interaction of dinitrogenase  [ **NIFK** ]   [ **NIFD** ]  reductase activating glycohydrolase DRAG with bacterial membranes and the solubilization of DRAG in response to nucleotides were characterized.  Ref: 10398752 Arch Microbiol, 1999
- Characterization of the interaction of dinitrogenase  [ **NIFK** ]   [ **NIFD** ]  reductase activating glycohydrolase from Rhodospirillum rubrum with bacterial membranes.  Ref: 10398752 Arch Microbiol, 1999
- Besides serving as the obligate electron donor to dinitrogenase  [ **NIFK** ]   [ **NIFD** ]  during nitrogenase turnover, dinitrogenase  [ **NIFK** ]   [ **NIFD** ]  reductase NifH is required for the biosynthesis of the iron molybdenum cofactor FeMo co and for the maturation of alpha 2 beta 2 apo dinitrogenase  [ **NIFK** ]   [ **NIFD** ]  apo dinitrogenase  [ **NIFK** ]   [ **NIFD** ]  maturation.  Ref: 10506203 J Biol Chem, 1999
- In Rhodospirillum rubrum, dinitrogenase  [ **NIFK** ]   [ **NIFD** ]  reductase ADP ribosyltransferase DRAT is responsible for the ADP ribosylation of dinitrogenase  [ **NIFK** ]   [ **NIFD** ]  reductase in response to the addition of NH 4 or removal from light, resulting in a decrease in nitrogenase activity.  Ref: 11160813 Microbiology, 2001

**Non-interactive relationship** (e.g. studied together, co-existance, homology, etc.)

- The adenylylation of GS plays no significant role in nif expression or the ADP ribosylation of dinitrogenase  [ **NIFK** ]   [ **NIFD** ]  reductase, since a mutant expressing GS Y398F showed normal nitrogenase activity and normal modification of dinitrogenase  [ **NIFK** ]   [ **NIFD** ]  reductase in response to NH 4 and darkness treatments.  Ref: 10648524 J Bacteriol, 2000
- The nifH operon nifH  **nifD**   **nifK**  has been reported previously.  Ref: 10217509 Microbiology, 1999
- Comparative analysis of the amino acid sequences of the four  **NifD** ,  **NifK** , NifE, and NifN in several archaeal and bacterial diazotrophs showed extensive sequence similarity between them, suggesting that their encoding genes constitute a novel paralogous gene family.  Ref: 10903367 J Mol Evol, 2000
- Certain Azotobacter vinelandii mutant strains unable to synthesize FeMo co accumulate an apo form of dinitrogenase  [ **NIFK** ]   [ **NIFD** ]  lacking FeMo co, with a subunit composition alpha 2 beta 2 gamma 2, which can be activated in vitro by the addition of FeMo co.  Ref: 11823455 J Biol Chem, 2002
- Through cross linking studies it was also shown that these changes decreased the ability of dinitrogenase  [ **NIFK** ]   [ **NIFD** ]  reductase to form a cross linkable complex with DRAT.  Ref: 10762264 J Bacteriol, 2000
- In certain bacteria switch off occurs by reversible covalent ADP ribosylation of the dinitrogenase  [ **NIFK** ]   [ **NIFD** ]  reductase protein, NifH.  Ref: 11208785 J Bacteriol, 2001
- As such this represents a new fold capable of binding FeMo co, where the only previous example was that seen in dinitrogenase  [ **NIFK** ]   [ **NIFD** ] .  Ref: 12754195 J Biol Chem< MedlineTA>J Biol Chem, 2001
- in addition, interrelationships between the control of known redox balancing systems, the Calvin Benson Bassham, dinitrogenase  [ **NIFK** ]   [ **NIFD** ]  and dimethyl sulfoxide reductase systems, were probed in strains grown under both photoheterotrophic and photoautotrophic growth conditions.  Ref: 11591679 J Bacteriol, 2001
- Dinitrogenase  [ **NIFK** ]   [ **NIFD** ]  is a heterotetrameric alpha 2 beta 2 enzyme that catalyzes the reduction of dinitrogen to ammonium and contains the iron molybdenum cofactor FeMo co at its active site.  Ref: 11823455 J Biol Chem, 2002
- The R101F and R101Y forms of dinitrogenase  [ **NIFK** ]   [ **NIFD** ]  reductase were able to form a complex with DRAT that could be chemically cross linked by 1 ethyl 3 3 dimethylaminopropyl carbodiimide.  Ref: 11114923 J Bacteriol, 2001
- Site directed mutagenesis of the draG gene was used to generate altered forms of dinitrogenase  [ **NIFK** ]   [ **NIFD** ]  reductase activating glycohydrolase DRAG with D123A, H142L, H158N, D243G, and E279R substitutions.  Ref: 11544238 J Bacteriol, 2001
- The nitrogenase regulating enzymes dinitrogenase  [ **NIFK** ]   [ **NIFD** ]  reductase ADP ribosyltransferase DRAT and dinitrogenase  [ **NIFK** ]   [ **NIFD** ]  reductase activating glycohydrolase DRAG, from Rhodospirillum rubrum, were shown to be sensitive to the redox status of the Fe 4 S 4 1 2 cluster of nitrogenase Fe protein from R. rubrum or Azotobacter vinelandii.  Ref: 10652344 J Biol Chem, 2000
- Reversible ADP ribosylation of dinitrogenase  [ **NIFK** ]   [ **NIFD** ]  reductase, catalyzed by the dinitrogenase  [ **NIFK** ]   [ **NIFD** ]  reductase ADP ribosyl transferase dinitrogenase  [ **NIFK** ]   [ **NIFD** ]  reductase activating glycohydrolase DRAT DRAG regulatory system, has been characterized in Rhodospirillum rubrum and other nitrogen fixing bacteria.  Ref: 11160092 J Bacteriol, 2001
- The strain containing the R101F form of dinitrogenase  [ **NIFK** ]   [ **NIFD** ]  reductase retains 91%, the strain containing the R101Y form retains 72%, and the strain containing the R101L form retains only 28% of in vivo nitrogenase activity of the strain containing the dinitrogenase  [ **NIFK** ]   [ **NIFD** ]  reductase with arginine at position 101.  Ref: 11114923 J Bacteriol, 2001
- IMS101 nifH,  **nifD**  and  **nifK**  amino acid sequences showed only 79%, 66% and 68% identity, respectively, to those of Anabaena sp.  Ref: 9884228 Microbiology, 1998
- Our dinitrogenase  [ **NIFK** ]   [ **NIFD** ]  gene nifH sequences grouped together with a group of cyanobacteria from the subtropical North Pacific.  Ref: 12406777 Appl Environ Microbiol, 2002
- The DNA isolated from the soil for this purpose was suitable for DNA DNA hybridization using 0.4 0.7 kb probes targeting denitrification enzymes, dinitrogenase  [ **NIFK** ]   [ **NIFD** ]  reductase nifH and eubacterial 16S rRNA.  Ref: 11377771 FEMS Microbiol Ecol< MedlineTA>FEMS Microbiol Ecol, 2002
- The production of dinitrogenase  [ **NIFK** ]   [ **NIFD** ]  reductase by GFP tagged bacteria was visualized using immunolocalization.  Ref: 10653751 Appl Environ Microbiol, 2000
- Role of the dinitrogenase  [ **NIFK** ]   [ **NIFD** ]  reductase arginine 101 residue in dinitrogenase  [ **NIFK** ]   [ **NIFD** ]  reductase ADP ribosyltransferase binding, NAD binding, and cleavage.  Ref: 11114923 J Bacteriol, 2001
- Thus, the Arg 140 residue of dinitrogenase  [ **NIFK** ]   [ **NIFD** ]  reductase plays a critical role in the ADP ribosylation reaction.  Ref: 10762264 J Bacteriol, 2000
- Altered dinitrogenase  [ **NIFK** ]   [ **NIFD** ]  reductases from strains UR629 R101Y and UR630 R101F were purified to homogeneity.  Ref: 11114923 J Bacteriol, 2001
- This altered form of NifH has been shown to inhibit substrate reduction by the wild type nitrogenase complex, forming a tight protein complex with dinitrogenase  [ **NIFK** ]   [ **NIFD** ] .  Ref: 10506203 J Biol Chem, 1999
- These results indicate that VnfY has a role in the maturation of the V dependent dinitrogenase  [ **NIFK** ]   [ **NIFD** ] , with a specific role in the formation of the V containing cofactor and or its insertion into apodinitrogenase.  Ref: 12644512 J Bacteriol, 2003
- In this study, mutant forms of dinitrogenase  [ **NIFK** ]   [ **NIFD** ]  reductase from A. vinelandii that are affected in various protein activities were tested for their ability to be ADP ribosylated or to form a complex with dinitrogenase  [ **NIFK** ]   [ **NIFD** ]  reductase ADP ribosyltransferase DRAT from Rhodospirillum rubrum.  Ref: 10762264 J Bacteriol, 2000
- Rhodospirillum rubrum strains in which the arginine 101 of dinitrogenase  [ **NIFK** ]   [ **NIFD** ]  reductase was replaced by tyrosine, phenylalanine, or leucine were constructed by site directed mutagenesis of the nifH gene.  Ref: 11114923 J Bacteriol, 2001
- Molecular characterization of Frankia strains using polymerase chain reaction restriction fragment length polymorphism PCR RFLP analyses of the 16S rRNA ITS gene and of the  **nifD**   **nifK**  spacer was conducted directly on root nodules collected worldwide from Casuarina and Allocasuarina trees.  Ref: 11207774 Environ Microbiol, 1999
- The MoFe protein component of the complex metalloenzyme nitrogenase is an alpha2beta2 tetramer encoded by the  **nifD**  and the  **nifK**  genes.  Ref: 12468552 J Biol Chem, 2003
- The biosynthesis of the iron molybdenum cofactor FeMo co of dinitrogenase  [ **NIFK** ]   [ **NIFD** ]  was investigated using 99Mo to follow the incorporation of Mo into precursors.  Ref: 10336491 J Biol Chem, 1999
- A mutant strain having an insertion mutation in vnfY has 10 fold less vnf dinitrogenase  [ **NIFK** ]   [ **NIFD** ]  activity and exhibits a greatly diminished level of 49 V label incorporation into the V dependent dinitrogenase  [ **NIFK** ]   [ **NIFD** ]  when compared to the wild type.  Ref: 12644512 J Bacteriol, 2003
- Little is known about the synthesis pathway of FeV co, other than that some of the gene products required are also involved in the synthesis of the iron molybdenum cofactor FeMo co of the widely studied molybdenum dinitrogenase  [ **NIFK** ]   [ **NIFD** ] .  Ref: 10364262 J Biol Chem, 1999
- Although restriction analysis of the  **nifD**   **nifK**  intergenic DNA fragment confirmed the unique nature of Rhizobium sp.  Ref: 12802480 Arch Microbiol< MedlineTA>Arch Microbiol, 1999
- We could not detect ADP ribosylation or any other covalent modification of dinitrogenase  [ **NIFK** ]   [ **NIFD** ]  reductase during switch off, suggesting that the mechanism differs from the well studied bacterial system.  Ref: 11208785 J Bacteriol, 2001
- Like DRAT WT, DRAT K103E also modified reduced dinitrogenase  [ **NIFK** ]   [ **NIFD** ]  reductase poorly.  Ref: 11160813 Microbiology, 2001
- Supporting this conclusion, we show that strains containing mutations in both nafY and nifX are severely affected in diazotrophic growth and extractable dinitrogenase  [ **NIFK** ]   [ **NIFD** ]  activity when cultured under conditions that are likely to occur in natural environments.  Ref: 11823455 J Biol Chem, 2002
- 99Mo label accumulates on dinitrogenase  [ **NIFK** ]   [ **NIFD** ]  only when all known components of the FeMo co synthesis system, NifH, NifNE, NifB cofactor, homocitrate, MgATP, and reductant, are present.  Ref: 10336491 J Biol Chem, 1999
- We also present evidence that the ability of NifH to function in these processes is not dictated by the properties known to be required for its function in electron transfer to dinitrogenase  [ **NIFK** ]   [ **NIFD** ] .  Ref: 10391920 J Biol Chem, 1999
- Both DRAT and dinitrogenase  [ **NIFK** ]   [ **NIFD** ]  reductase can be labeled by carbonyl 14 C NAD individually upon UV irradiation, but most 14 C label is incorporated into DRAT when both proteins are present.  Ref: 11114923 J Bacteriol, 2001
- Structural analogues of homocitrate prevent the acetylene reduction ability of the resulting dinitrogenase  [ **NIFK** ]   [ **NIFD** ] .  Ref: 11053414 J Biol Chem, 2001
- Effects of specific amino acid substitutions on activities of dinitrogenase  [ **NIFK** ]   [ **NIFD** ]  reductase activating glycohydrolase from Rhodospirillum rubrum.  Ref: 11544238 J Bacteriol, 2001
- A portion of the dinitrogenase  [ **NIFK** ]   [ **NIFD** ]  reductase gene nifH was directly amplified from DNA extracted from the mixed population in the termite gut.  Ref: 10543805 Appl Environ Microbiol, 1999
- This suggests that arginine 101 is not critical for the binding of DRAT to dinitrogenase  [ **NIFK** ]   [ **NIFD** ]  reductase but that the availability of arginine 101 is important for NAD cleavage.  Ref: 11114923 J Bacteriol, 2001
- The diminished rates of substrate reduction were found to correlate with the decreased amounts of the dinitrogenase  [ **NIFK** ]   [ **NIFD** ]  component MoFe protein expressed in R. capsulatus.  Ref: 11277916 Eur J Biochem, 2001
- These symbionts are thought to provide enzymes, e.g., cellulase and dinitrogenase  [ **NIFK** ]   [ **NIFD** ] , which assist the host in utilizing wood as a primary food source.  Ref: 12450854 Appl Environ Microbiol, 2002
- The ability of the isolates to fix dinitrogen was evaluated by amplification of the  **nifD**  gene, immunodetection of the dinitrogenase  [ **NIFK** ]   [ **NIFD** ]  reductase and acetylene reduction assay.  Ref: 11211255 Int J Syst Evol Microbiol, 2001
- Analysis revealed three open reading frames encoding putative proteins with a very high degree of sequence similarity with the two subunits  **NifD**  and  **NifK**  of the component I and with component II NifH of nitrogenase from different diazotrophs.  Ref: 11157237 Appl Environ Microbiol, 2001
- Significant amounts of both dinitrogenase  [ **NIFK** ]   [ **NIFD** ]  and dinitrogenase  [ **NIFK** ]   [ **NIFD** ]  reductase were only formed when the growth medium was supplemented with molybdate 1 microM.  Ref: 12667075 Biochemistry, 2003
- IMS101 reveals stable  **nifD** , nifDK and  **nifK**  transcripts.  Ref: 9884228 Microbiology, 1998
- In contrast to previous studies with other organisms, the dinitrogenase  [ **NIFK** ]   [ **NIFD** ]  reductase polypeptides were maintained in an active and unmodified form in strain SBP PHC and the respective RCNd strains.  Ref: 11131022 Arch Microbiol, 2000
- These mutants showed lower nitrogenase activity and a higher degree of ADP ribosylation of dinitrogenase  [ **NIFK** ]   [ **NIFD** ]  reductase under N 2 fixing conditions than was seen in a wild type control strain.  Ref: 11160813 Microbiology, 2001
- In contrast, a mutant with an insertion in draG was still capable of ADP ribosylating dinitrogenase  [ **NIFK** ]   [ **NIFD** ]  reductase in response to ammonium but was no longer able to recover activity after ammonium depletion.  Ref: 10935160 Chin J Biotechnol, 1999
- Effect of P II and its homolog GlnK on reversible ADP ribosylation of dinitrogenase  [ **NIFK** ]   [ **NIFD** ]  reductase by heterologous expression of the Rhodospirillum rubrum dinitrogenase  [ **NIFK** ]   [ **NIFD** ]  reductase ADP ribosyl transferase dinitrogenase  [ **NIFK** ]   [ **NIFD** ]  reductase activating glycohydrolase regulatory system in Klebsiella pneumoniae.  Ref: 11160092 J Bacteriol, 2001
- Molecular evolution of nitrogen fixation the evolutionary history of the  **nifD** ,  **nifK** , nifE, and nifN genes.  Ref: 10903367 J Mol Evol, 2000
- The dra operon encodes dinitrogenase  [ **NIFK** ]   [ **NIFD** ]  reductase ADP ribosyltransferase and dinitrogenase  [ **NIFK** ]   [ **NIFD** ]  reductase activating glycohydrolase, enzymes responsible for the reversible inactivation, via ADP ribosylation, of nitrogenase Fe protein in R. rubrum.  Ref: 10850982 J Bacteriol, 2000
- In vitro synthesis of FeMo co has been established, and the reaction requires an ATP regenerating system, dithionite, molybdate, homocitrate, and at least NifB co the metabolic product of NifB, NifNE, and dinitrogenase  [ **NIFK** ]   [ **NIFD** ]  reductase NifH.  Ref: 10217770 J Bacteriol, 1999
- In contrast, either GlnB or GlnJ can serve as a critical element in regulation of the reversible ADP ribosylation of dinitrogenase  [ **NIFK** ]   [ **NIFD** ]  reductase catalyzed by the dinitrogenase  [ **NIFK** ]   [ **NIFD** ]  reductase ADP ribosyl transferase DRAT dinitrogenase  [ **NIFK** ]   [ **NIFD** ]  reductase activating glycohydrolase DRAG regulatory system.  Ref: 11591658 J Bacteriol, 2001
- The dinitrogenase  [ **NIFK** ]   [ **NIFD** ]  component proteins of the conventional Mo nitrogenase MoFe protein and of the alternative Fe only nitrogenase FeFe protein were both isolated and purified from Rhodobacter capsulatus, redox titrated according to the same procedures and subjected to an EPR spectroscopic comparison.  Ref: 11895435 Eur J Biochem, 2002
- ADP Ribosylation of variants of Azotobacter vinelandii dinitrogenase  [ **NIFK** ]   [ **NIFD** ]  reductase by Rhodospirillum rubrum dinitrogenase  [ **NIFK** ]   [ **NIFD** ]  reductase ADP ribosyltransferase.  Ref: 10762264 J Bacteriol, 2000
- Fragments of the nifH gene, which encodes the dinitrogenase  [ **NIFK** ]   [ **NIFD** ]  reductase, were directly amplified from the mixed population DNA of the termite gut and were clonally isolated.  Ref: 9783160 Extremophiles, 1998
- Moreover, dinitrogenase  [ **NIFK** ]   [ **NIFD** ]  derepression in Rba.  Ref: 11131022 Arch Microbiol, 2000
- Previously, the proton reducing activity of the dinitrogenase  [ **NIFK** ]   [ **NIFD** ]  complex was implicated in helping to maintain redox balance.  Ref: 10763751 Arch Microbiol, 2000
- R140Q dinitrogenase  [ **NIFK** ]   [ **NIFD** ]  reductase could not be ADP ribosylated by DRAT, although it still formed a cross linkable complex with DRAT.  Ref: 10762264 J Bacteriol, 2000
- Several DRAT variants that are altered both in the posttranslational regulation of DRAT activity and in the ability to recognize variants of dinitrogenase  [ **NIFK** ]   [ **NIFD** ]  reductase have been found.  Ref: 10049407 J Bacteriol, 1999
- In a number of nitrogen fixing bacteria, nitrogenase is posttranslationally regulated by reversible ADP ribosylation of dinitrogenase  [ **NIFK** ]   [ **NIFD** ]  reductase.  Ref: 10762264 J Bacteriol, 2000
- Samples of the dithionite reduced FeFe protein the dinitrogenase  [ **NIFK** ]   [ **NIFD** ]  component of the Fe only nitrogenase from Rhodobacter capsulatus have been investigated by 57Fe Mössbauer spectroscopy and by Fe and Zn EXAFS as well as XANES spectroscopy.  Ref: 11862539 J Biol Inorg Chem, 2002
- The R101F form of dinitrogenase  [ **NIFK** ]   [ **NIFD** ]  reductase and DRAT together were not able to cleave NAD.  Ref: 11114923 J Bacteriol, 2001
- Regulation of dinitrogenase  [ **NIFK** ]   [ **NIFD** ]  reductase ADP ribosyltransferase and dinitrogenase  [ **NIFK** ]   [ **NIFD** ]  reductase activating glycohydrolase by a redox dependent conformational change of nitrogenase Fe protein.  Ref: 10652344 J Biol Chem, 2000
- In specific mutant strains of R. capsulatus, expression of both the Calvin Benson Bassham and dinitrogenase  [ **NIFK** ]   [ **NIFD** ]  systems was influenced by dimethyl sulfoxide respiration.  Ref: 11591679 J Bacteriol, 2001
- Apart from serving as the physiological electron donor to dinitrogenase  [ **NIFK** ]   [ **NIFD** ] , NifH is involved in iron molybdenum cofactor FeMo co biosynthesis and in maturation of the FeMo co deficient form of apodinitrogenase to a FeMo co activable form apodinitrogenase maturation.  Ref: 10391920 J Biol Chem, 1999
- As another level of control, nitrogenase activity was regulated by at least three types of mechanisms in strain BH72 covalent modification of dinitrogenase  [ **NIFK** ]   [ **NIFD** ]  reductase NifH, probably by ADP ribosylation, and two other, unknown means.  Ref: 11914357 J Bacteriol, 2002
- The Fe only nitrogenase and the Mo nitrogenase from Rhodobacter capsulatus a comparative study on the redox properties of the metal clusters present in the dinitrogenase  [ **NIFK** ]   [ **NIFD** ]  components.  Ref: 11895435 Eur J Biochem, 2002
- The key nitrogen fixation nif genes, including nifH,  **nifD** , and  **nifK**  for nitrogenase component proteins as well as nifE, nifN, nifB and nifV for synthesis of the iron molybdenum cofactor FeMoco of nitrogenase, have now been identified in C. acetobutylicum or C. beijerinckii or both.  Ref: 11781802 J Ind Microbiol Biotechnol, 2001
- VnfY is required for full activity of the vanadium containing dinitrogenase  [ **NIFK** ]   [ **NIFD** ]  in Azotobacter vinelandii.  Ref: 12644512 J Bacteriol, 2003
- However, in in vitro measurements with cell free extracts as well as purified dinitrogenase  [ **NIFK** ]   [ **NIFD** ] , ethane production was always found to be less than 1%.  Ref: 11277916 Eur J Biochem, 2001
- Nitrogen fixation is tightly regulated in Rhodospirillum rubrum at two different levels transcriptional regulation of nif expression and posttranslational regulation of dinitrogenase  [ **NIFK** ]   [ **NIFD** ]  reductase by reversible ADP ribosylation catalyzed by the DRAT DRAG dinitrogenase  [ **NIFK** ]   [ **NIFD** ]  reductase ADP ribosyltransferase dinitrogenase  [ **NIFK** ]   [ **NIFD** ]  reductase activating glycohydrolase system.  Ref: 10648524 J Bacteriol, 2000
- Clear distinction between Sinorhizobium meliloti and Sinorhizobium medicae were observed by analysis of the IGS symbiotic regions between  **nifD**  and  **nifK**  genes.  Ref: 11261493 Can J Microbiol, 2001
- Surprisingly, Northern hybridization also revealed the presence of transcripts that corresponded to  **nifD** , nifDK and  **nifK** , which have not been previously reported as transcripts in contiguous nifHDK genes of cyanobacteria.  Ref: 9884228 Microbiology, 1998
- Under salt stress, the amount of dinitrogenase  [ **NIFK** ]   [ **NIFD** ]  reductase inactivated by ADP ribosylation was strongly reduced, indicating that the dinitrogenase  [ **NIFK** ]   [ **NIFD** ]  reductase ADP ribosyl transferase DRAT activity was also inhibited by increased NaCl concentrations.  Ref: 11927988 Curr Microbiol, 2002
- The seven representatives of BOX PCR types produced indole acetic acid, reduced acetylene and showed specific immunological cross reaction with anti dinitrogenase  [ **NIFK** ]   [ **NIFD** ]  reductase antibody.  Ref: 11566385 J Biotechnol, 2001
- The structure of the dinitrogenase  [ **NIFK** ]   [ **NIFD** ]  reductase from Azotobacter vinelandii is known.  Ref: 10762264 J Bacteriol, 2000
- Isolation and characterization of draT mutants that have altered regulatory properties of dinitrogenase  [ **NIFK** ]   [ **NIFD** ]  reductase ADP ribosyltransferase in Rhodospirillum rubrum.  Ref: 11160813 Microbiology, 2001
- Immunolocalization of dinitrogenase  [ **NIFK** ]   [ **NIFD** ]  reductase produced by Klebsiella pneumoniae in association with Zea mays L.  Ref: 10653751 Appl Environ Microbiol, 2000
- Previous results showed that changing Lys 143 to Gln decreased the binding between dinitrogenase  [ **NIFK** ]   [ **NIFD** ]  reductase and dinitrogenase  [ **NIFK** ]   [ **NIFD** ]  L.  Ref: 10762264 J Bacteriol, 2000
- Substitution of D129E or deletion of Leu 127, which result in altered nucleotide binding regions of these dinitrogenase  [ **NIFK** ]   [ **NIFD** ]  reductases, did not significantly change the interaction between dinitrogenase  [ **NIFK** ]   [ **NIFD** ]  reductase and DRAT.  Ref: 10762264 J Bacteriol, 2000
- Conformational changes in dinitrogenase  [ **NIFK** ]   [ **NIFD** ]  reductase induced by an F135Y substitution or by removal of the Fe 4 S 4 cluster resulted in dinitrogenase  [ **NIFK** ]   [ **NIFD** ]  reductase not being a substrate for ADP ribosylation.  Ref: 10762264 J Bacteriol, 2000
- In vivo acetylene reduction assays, immunoblotting with anti dinitrogenase  [ **NIFK** ]   [ **NIFD** ]  reductase antibody, and adenylate 32 P NAD labeling experiments showed that no switch off of nitrogenase activity occurred in any of the three mutants and no ADP ribosylation of altered dinitrogenase  [ **NIFK** ]   [ **NIFD** ]  reductases occurred either in vivo or in vitro.  Ref: 11114923 J Bacteriol, 2001
- In all diazotrophic micro organisms investigated so far, mutations in nifE, one of the genes involved in the biosynthesis of the FeMo cofactor FeMoco, resulted in the accumulation of cofactorless inactive dinitrogenase  [ **NIFK** ]   [ **NIFD** ] .  Ref: 11277916 Eur J Biochem, 2001
- This regulation is mediated by two enzymes, dinitrogenase  [ **NIFK** ]   [ **NIFD** ]  reductase ADP ribosyl transferase DRAT and dinitrogenase  [ **NIFK** ]   [ **NIFD** ]  reductase activating glycohydrolase DRAG.  Ref: 11315111 Can J Microbiol, 2001
- In Rhodospirillum rubrum, nitrogenase activity is regulated posttranslationally through the ADP ribosylation of dinitrogenase  [ **NIFK** ]   [ **NIFD** ]  reductase by dinitrogenase  [ **NIFK** ]   [ **NIFD** ]  reductase ADP ribosyltransferase DRAT.  Ref: 10049407 J Bacteriol, 1999
- Mutational analyses suggested that FdxN is an important but not the essential electron donor for dinitrogenase  [ **NIFK** ]   [ **NIFD** ]  reductase.  Ref: 11371540 J Bacteriol, 2001
- The ability of R101F dinitrogenase  [ **NIFK** ]   [ **NIFD** ]  reductase to be labeled by carbonyl 14 C NAD suggested that Arg 101 is not absolutely required for NAD binding.  Ref: 11114923 J Bacteriol, 2001
- The endophytic lifestyle of Klebsiella pneumoniae is described, including the production of dinitrogenase  [ **NIFK** ]   [ **NIFD** ]  reductase by bacteria residing in maize root tissue.  Ref: 10653751 Appl Environ Microbiol, 2000
- These strains SBP PHC and RCNd1, RCNd3, and RCNd4 were shown to obviate normal ammonia control and derepress synthesis of the dinitrogenase  [ **NIFK** ]   [ **NIFD** ]  enzyme complex for the dissipation of excess reducing equivalents and generation of H2 gas via proton reduction.  Ref: 11131022 Arch Microbiol, 2000
- Another novel S = 1 2 signal at g = 1.9.92 and 1.77 was observed on further, enzymatic reduction of the dithionite reduced state of Rc1Fe with the dinitrogenase  [ **NIFK** ]   [ **NIFD** ]  reductase component Rc2Fe of the same enzyme system turnover conditions in the presence of N2 and ATP.  Ref: 11895435 Eur J Biochem, 2002
